# The Bacterial Anti-Adhesive Activity of Double-Etched Titanium (DAE) as a Dental Implant Surface

**DOI:** 10.3390/ijms21218315

**Published:** 2020-11-05

**Authors:** Morena Petrini, Alessandra Giuliani, Emanuela Di Campli, Silvia Di Lodovico, Giovanna Iezzi, Adriano Piattelli, Simonetta D’Ercole

**Affiliations:** 1 Department of Medical, Oral and Biotechnological Science, University “G. d’Annunzio” Chieti –Pescara, Via dei Vestini 31, 66013 Chieti, Italy; morena.petrini@unich.it (M.P.); gio.iezzi@unich.it (G.I.); simonetta.dercole@unich.it (S.D.); 2 Department of Clinical Sciences, Polytechnic University of Marche, Via Brecce Bianche, 60100 Ancona, Italy; a.giuliani@univpm.it; 3 Department of Pharmacy, University “G. d’Annunzio” Chieti –Pescara, Via dei Vestini 31, 66013 Chieti, Italy; edicampli@unich.it (E.D.C.); silvia.dilodovico@unich.it (S.D.L.); 4 Biomaterials Engineering, Catholic University of San Antonio de Murcia (UCAM), Av. de los Jerónimos, Guadalupe, 135 30107 Murcia, Spain; 5 Fondazione Villa Serena per la Ricerca, Città S. Angelo, Via Petruzzi 42, 65013 Pescara, Italy

**Keywords:** titanium, *Streptococcus oralis*, biofilms, dental implants, microscopy, atomic force, microscopy, electron, scanning, wettability, peri-implantitis

## Abstract

This work aimed to compare the capability of *Streptococcus oralis* to adhere to a novel surface, double-etched titanium (DAE), in respect to machined and single-etched titanium. The secondary outcome was to establish which topographical features could affect the interaction between the implant surface and bacteria. The samples’ superficial features were characterized using scanning electron microscopy (SEM) and energy dispersive x-ray spectrometry (EDS), and the wetting properties were tested through sessile methods. The novel surface, the double-etched titanium (DAE), was also analyzed with atomic force microscopy (AFM). *S. oralis* was inoculated on discs previously incubated in saliva, and then the colony-forming units (CFUs), biomass, and cellular viability were measured at 24 and 48h. SEM observation showed that DAE was characterized by higher porosity and Oxygen (%) in the superficial layer and the measurement of the wetting properties showed higher hydrophilicity. AFM confirmed the presence of a higher superficial nano-roughness. Microbiological analysis showed that DAE discs, coated by pellicle’s proteins, were characterized by significantly lower CFUs at 24 and 48 h with respect to the other two groups. In particular, a significant inverse relationship was shown between the CFUs at 48 h and the values of the wetted area and a direct correlation with the water contact angle. The biomass at 24 h was slightly lower on DAE, but results were not significant concerning the other groups, both at 24 and 48 h. The DAE treatment not only modifies the superficial topography and increased hydrophilicity, but it also increases the Oxygen percentage in the superficial layer, which could contribute to the inhibition of *S. oralis* adhesion. DAE can be considered a promising treatment for titanium implants to counteract a colonization pioneer microorganism, such as *S. oralis.*

## 1. Introduction

### 1.1. Background

The study of variables that could affect the early and late failures of dental implants is fundamental for developing useful tools to prevent or treat the disease. Superficial treatments of dental implants have been shown to increase the surface in contact with the bone, promote osseointegration, accelerate the loading protocols, and decrease early failure rates [1,2]. Indeed, the peri-implant zone is an active environment of ~1 mm in width. The interactions between the implant and the bone are continuously mediated by cell-signaling pathways (such as the Wnt-β catenin pathway), influencing the bone remodeling process [3]. In particular, superficial micro-porosities, increasing titanium’s wettability, can affect cellular adhesion, diffusion, migration, proliferation, and differentiation [4,5,6].

Many superficial treatments, mechanical, chemical, and physical, have been described to increase superficial roughness, optimize the healing after the implant insertion, shorten the time necessary to obtain osseointegration, and promote the long-term stability of hard and soft tissues [7]. Additive methods comprise the implant coating with biomaterials, such as hydroxyapatite (HA) and calcium phosphate (CaP) with micro or nano-coatings. Subtractive techniques can be sandblasting with abrasives, such as TiO2, AlO2, hydroxyapatite (HA), laser, electrochemical deposition, acid-etching, or double acidification, or a combination of some of these treatments. In recent years, a novel surface characterized by a double etching (DAE) has been produced. This method consists of a twice treatment of samples previously ultrasonicated with a solution mix containing nitric, hydrofluoric, and hydrochloric acids, followed by a neutralizing buffer and a final washing with a basic solution in an ultrasonic bath. Then, at the end of the treatment, the samples are decontaminated in an argon cold plasma reactor.

The aim of DAE treatment is to introduce an overlapped roughness in the nanometer to the submicron range [8]. Giner et al. [8,9] have studied a titanium surface sandblasted and then double-etched: under SEM, a structure similar to the lacunae created by osteoclasts during bone resorption (Howship’s lacunae) [8,9] was observed. They found that double-etching treatment increased the adhesion and proliferation of the osteoblast-like cell line with respect to controls significantly, and alkaline phosphatase (ALP) was characterized by a six-fold increase on day 7. However, it has also been shown that despite lower early failure rates, the treated surface could increase microbial adhesion and make more difficult the oral hygiene maneuvers and professional decontamination [1]. Coelho et al. [10] suggested that implants with increased surface roughness were the ideal solution in the case of low bone density; microbial adhesion in case of surface colonization could, however, be encouraged.

Bacterial colonization of the dental implant begins directly after exposure to the oral environment. Microbiota can be identified 30 minutes after implant installation and within two weeks, with an established community similar to that found around natural teeth in the same mouth [11]. Roehling et al. [12] believe that a reduced bacterial adhesion on implant surfaces might be clinically associated with a reduced risk or incidence of peri-implant infections. Several factors affect this process, e.g., bacterial properties and material surface characteristics. The hypothesis is that increased roughness and wettability could favor bacterial adhesion, increasing the risk of peri-implantitis. The characterization of machined and etched surfaces with atomic force microscopy (AFM) and the ability of different bacteria to form a biofilm on these surfaces has been largely analyzed in literature [13,14,15,16,17]. However, there is not a total consensus about this topic in literature, and contrasting results have been shown [12,16]. *Streptococcus oralis* is an initial colonizer in human plaque. It adheres to the acquired pellicle of soft and hard tissues in the oral cavity, creating preconditions for the adhesion of late colonizers [18]. Then, the adherence, proliferation, and biofilm formation of this bacterium enhance the colonization of other pathogens; these considerations lead to the inclusion of *Streptococcus spp.* in the Venn diagram of the microbiome distribution in peri-implantitis [19].

### 1.2. Aim

The aim of this work is to compare the capability of *Streptococcus oralis* to adhere to DAE with respect to machined and single-etched discs. The second outcome is to understand which topographical parameter could affect bacterial interaction with the implant surface.

## 2. Results

**Figure 1:**
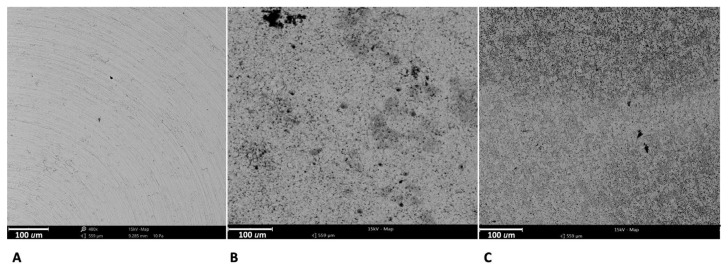
The superficial micro-topography observed at SEM of machined discs (**A**), single-etched discs (**B**), and double-etched (DAE) ones (**C**). The magnification is 480×.

The SEM observation at 480× (Figure 1) shows the microscopical features of the samples: machined surfaces (Figure 1A) were characterized by a very regular structure, characterized by the presence of circumferential and parallel lines. The acidified surfaces (Figure 1B,C) are characterized by a porous structure composed of the random arrangement of furrows of various sizes.

At this magnification, it is possible to observe how the DAE surface (Figure 1C) is characterized by a greater quantity of pores, smaller in size compared to group 2 (Figure 1B). 

**Figure 2:**
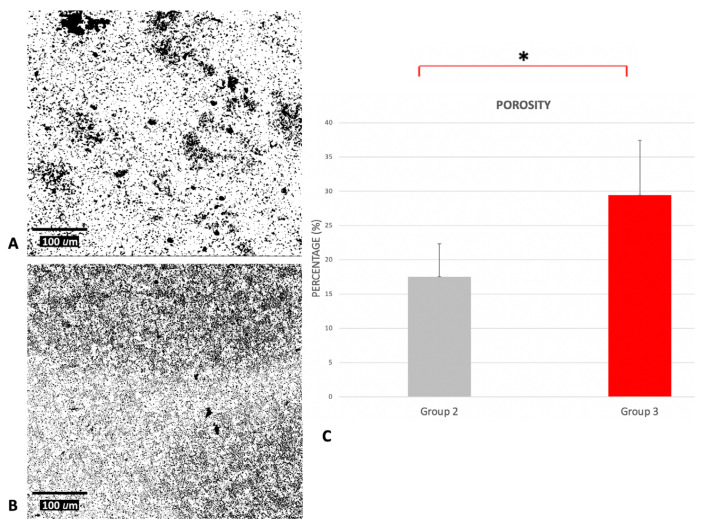
SEM images of etched (**A**) and DAE (**B**) samples at 480× magnification after the process of binarization that permitted the measurement of the percentage of porosity by using ImageJ 1.52q for Mac OS X (USA). (**C**) Average values of the percentage of porosity of the samples (error bars = standard deviation), calculated on 10 different pictures for each group. * *p*-value < 0.01.

The calculated percentage of porosity, Figure 2, was significantly higher (*p* < 0.001) on DAE samples (29.422% ± 8.000), with respect to etched ones (17.545% ± 4.778). The Pearson analysis showed that the percentage of porosity was directly correlated with the wetted area (*r* = 0.861, *p* < 0.01) and inversely with the water contact angle (WCA) (*r* = −0.906, *p* < 0.01).

**Figure 3:**
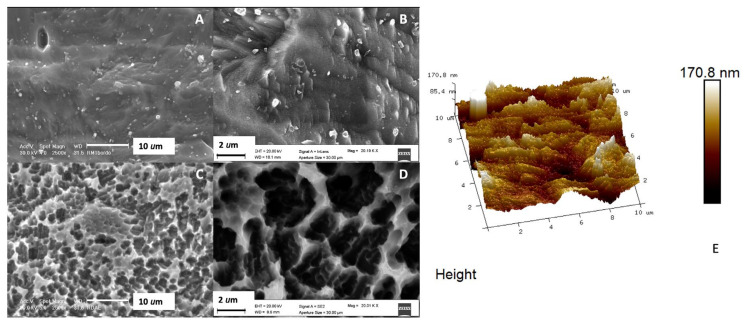
SEM images of titanium discs etched (group 2), 2500× (**A**), high magnification 20K× (**B**) and (group 3) DAE discs, 2500× (**C**) and high magnification 20K× (**D**). (**E**) 3D reconstruction of atomic force microscope (AFM) images (10um × 10um) of DAE discs (group 3).

Increasing the magnification to 2500× (Figure 3), group 2 (Figure 3A) appeared minimally rough at the micrometric level. Few pores of big dimensions, 2−4 μm in diameter, were detected together with sparse grain clusters, not uniformly distributed in the sample, and with variable dimensions (from 200−300 nanometers to 2−4 μm). At a higher magnification, 20K× (Figure 3B), the surface appeared rougher, with a rugosity apparently uniformly distributed in the sample and with variable dimensions of 50−100 nanometers.

DAE surfaces appeared highly rough at 2500× (Figure 3C), several pores with dimensions varying from 200−400 nm to 2−4 μm in diameter were observed. Very few grain clusters (with small dimensions) were detected, most likely vanishing after the second etching phase. At 20K× magnification (Figure 3D), the sample surface did not reveal a smaller porosity than 200 nm.

Atomic force microscopy (AFM) observation of DAE samples confirmed the presence of nano-roughness and a roughness average (Ra) of 28.957 nm (± 8.856), calculated in an observation area of 10 µm × 10 µm (Figure 3E). 

The energy dispersive x-ray spectrometry (EDS) analysis at 15 kV has shown that the chemical composition of the three samples was very similar at deeper layers; indeed, all samples were characterized by a weight concentration of TiO_2_ comprised between 92% and 93% and La_2_O_3_ between 7% and 8%, without any significant differences among groups.

**Figure 4:**
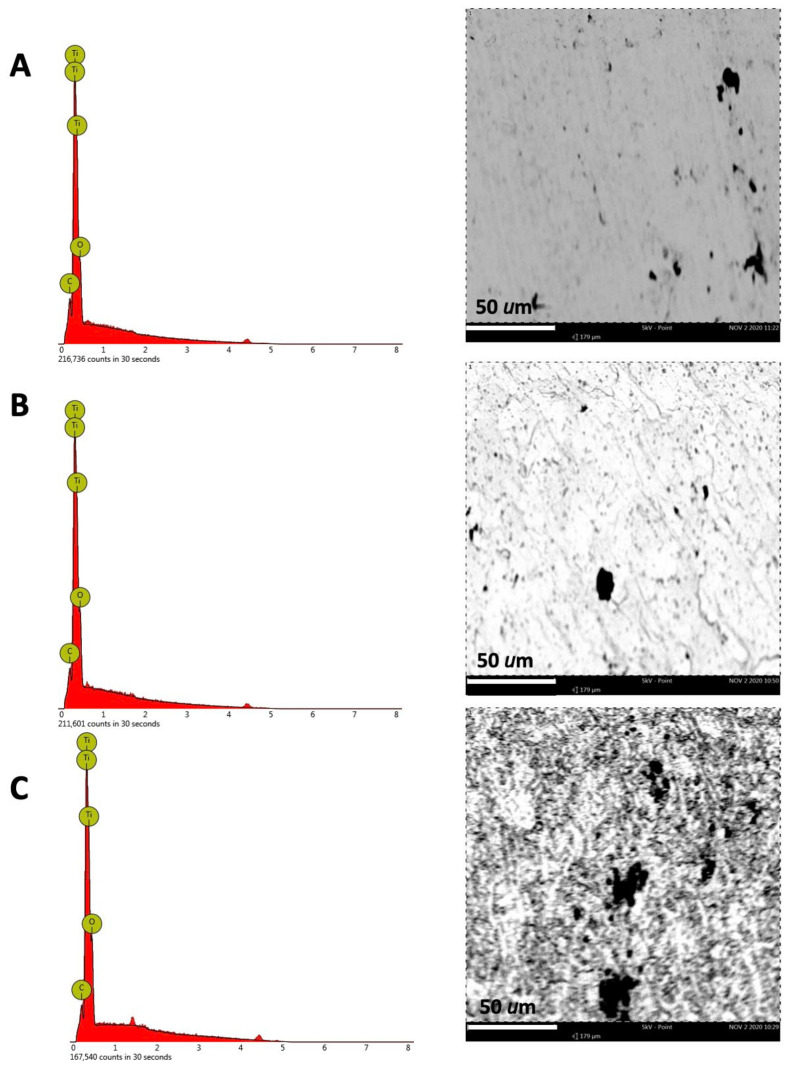
Spectra of energy dispersive x-ray spectrometry (EDS) analysis at 5 kV of titanium discs machined, **A**, (group 1), etched, **B**, (group 2), and DAE, **C**, (group 3) with the relative picture of the surface analyzed at 1500×.

On the contrary, at 5 kV, a significant difference in the percentage of titanium and Oxygen was found; Figure 4, Table 1. A higher percentage of O characterized the DAE samples (5.05% ± 0.713), followed by etched (4.323% ± 0.166) and machined (3.723% ± 0.202). ANOVA and the least significant difference (LSD) post hoc test found significant differences (*p* < 0.05) between machined and DAE for all parameters investigated.

**Figure 5:**
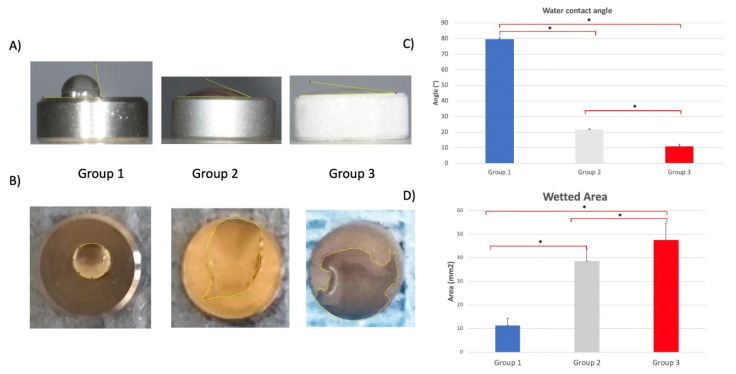
(**A**) Static contact angles with sessile drop method and (**B**) wetted area of groups 1, 2, and 3. (**C**) Average water contact angle. * *p*-value < 0.05. (**D**) Average wetted area (error bars = standard deviation) * *p*-value < 0.05.

Measurement of the wetting properties (Figure 5) has shown that all surfaces could be considered as hydrophilic because they were characterized by a contact angle smaller than 90°. However, both contact angles (Figure 5A,C) and wetted area (Figure 5B,D) measurements have shown that DAE surfaces (Group 3) were characterized by significantly increased hydrophilicity with respect to the other two groups, followed by group 2 (*p* < 0.05).

**Figure 6:**
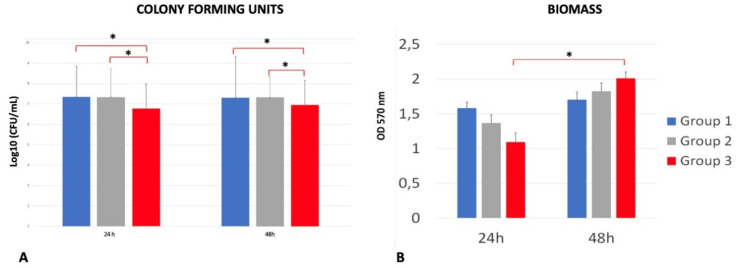
Colony-forming units (log10)/mL (**A**) and biofilm biomass formation (**B**) of *S. oralis* on titanium discs machined (group 1), etched (group 2) and double-etched (group 3) at 24 and 48 h (error bars = standard deviation). *p-value<0.05.

The results of the colony-forming units are shown in Figure 6A. The number of colony-forming units (CFU)/mL was significantly lower in the DAE group when compared to group 1 (*p* < 0.001) and group 2 (*p* = 0.001) at 24 h. There were no significant differences when comparing group 1 and group 2. A similar trend was found at 48 h: group 3 was characterized by a significantly lower density of CFUs when compared to group 1 (*p* = 0.029) and group 2 (*p* = 0.020). No differences were detected between groups 1 and 2. Figure 6B shows the biomass produced by *S. oralis* on the analyzed surfaces. DAE discs showed lower values after 24 h of incubation, but the results were not significant when compared to the other groups. 

**Figure 7:**
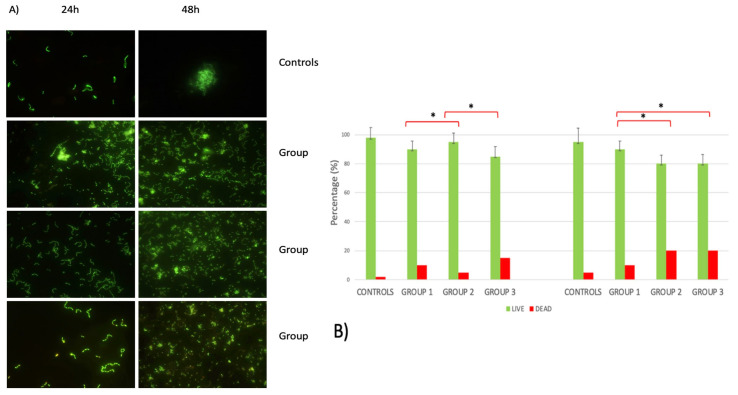
(**A**) Live/dead images of *S. oralis* on controls and groups 1, 2, and 3 at 24 and 48 h (green: live cells; red: dead cells). (**B**) Percentage of living cells at live/dead observation. * *p*-value < 0.05.

The inter-group analysis showed no significant differences also at 48 h, but it is interesting to highlight a significant increase in DAE biofilm mass between 24 and 48 h (*p* = 0.001). Live/dead analysis (Figure 7) showed bacterial cells grouped in microcolonies and a predominance of live bacteria with respect to the dead ones in all samples.

As the incubation time progressed to 48 h, the biofilm structure acquired a more complex morphology and an increased biofilm when compared to 24 h. The DAE group discs were characterized by a lower capability in *S. oralis* attractiveness and organization in biofilm than the other surfaces. The percentage of live cells in the DAE group was lower at 24 h when compared to the other groups, but at 48 h, the results were similar between the etched groups.

Group 2 and group 3 were characterized by a lower percentage of live cells when compared to group 1, both at 24 and 48 h. The results of the Pearson analysis are shown in Table 2; a significant inverse relationship was shown between the CFUs at 48 h and the values of the wetted area (*r* = −0.737, *p* = 0.023) and a direct correlation with the water contact angle (WCA) (*r* = 0.778, *p* = 0.013).

## 3. Discussion

Peri-implantitis is a condition that is very difficult to treat, and for this reason, research studies are trying to find alternative methods of treatment and searching for novel materials and surfaces that could decrease bacterial adhesion [20,21,22,23,24,25,26,27]. The functionalization of polymeric materials is a promising strategy for the production of surfaces with antimicrobial activity [28].

The objective of this study was to compare the capability of *Streptococcus oralis* to adhere to a novel surface, the titanium double-etched (DAE) surface, with respect to machined and single-etched titanium. The secondary outcome was to compare their superficial features in order to better understand their relationship with bacteria.

The double acid-etching surface is labeled by higher wettability and cellular adhesion, with increased bacterial proliferation [29,30]. A recent study has shown that DAE discs are characterized by increased cell growth, cell adhesion, and improved osteogenic and angiogenic events, as well as the osseointegration process, with respect to machined samples [31]. AFM demonstrated that the double etching treatment enhanced the formation of a nano-roughness surface, and average roughness Ra remained under 0.2 µm.

Nano-roughness is fundamental for the increase in protein adsorption and the modulation of cell signaling [8,30]. 

In this study, the double acid-etched discs, at the SEM observation, showed an increased porosity with respect to the single-etched surface, with pores of a measure comprised between 200 nm to 4 µm. The wetting properties measurements showed that DAE surfaces were characterized by higher hydrophilicity with respect to other groups.

On the contrary, as shown in literature, machined and single-etched surfaces are characterized by higher values of Ra, greater than 0.3 µm [13]. 

For microbiological analysis, we chose to use *S. oralis*, a Gram-positive and facultative anaerobic bacterium, because it is one of the first pioneers that colonizes teeth and dental implants in vivo [18]. It is an opportunistic pathogen, and the adherence, proliferation, and biofilm formation of this bacteria create preconditions for adhesion of other pathogens, such as *Porphyromonas gingivalis,* that have also been implicated with the onset of other chronic systemic diseases [19,32]. 

In order to permit the formation of acquired pellicle on the discs and obtain a condition more similar to the oral cavity, the titanium surfaces were incubated with saliva, and the bacteria were inoculated and left to produce the biofilm for 24, and 48 h. *S. oralis* was able to adhere to titanium surfaces covered by an acquired pellicle by the expression of adhesins that bind multiple salivary proteins, including MUC7, proline-rich proteins, and amylase [33]. Surprisingly, after 24 and 48 h, the number of CFUs on DAE discs was significantly lower when compared to other surfaces. A similar trend was also found for biomass quantification at 24 h but without statistically significant differences among the groups. On the contrary, there were no differences between titanium discs with machined and single-etched surfaces. These results were more interesting because DAE discs were characterized by the presence of higher porosity and hydrophilicity with respect to other groups.

These results are very encouraging for the clinical setting because if we consider that domiciliary oral hygiene maneuvers for the intact tooth should be performed twice a day by means of toothbrushes and interdental devices, this type of surface that contrasts biofilm accumulation in the first hours after plaque removal could be of valid help to maintain the hygiene level around implants [34,35].

There is disagreement in the literature about the influence of the topographical configuration of titanium on the ability of bacteria to proliferate and organize a biofilm. Amoroso et al. [36] showed that a superficial Ra below 0.2 μm does not promote microbial adherence because most bacteria are larger than this size. However, another parameter that influences results is the timing point; the interaction between bacteria and surfaces is initiated by non-specific forces and is then mediated by specific binding between a bacterium and substrate [37,38]. Consequently, the topographical conformation of titanium exerts its influence mainly in the first steps of bacterial colonization. Schmidlin et al. [15] concluded that superficial roughness was able to influence bacterial adhesion only in the first hours, but after the establishment of the biofilm that they quantified occurring after 16.5 h, the differences between the groups were not significant. In the present study, the positive effects of DAE surfaces in maintaining significantly lower levels of CFUs were confirmed both at 24 and at 48 h, although on the first day, the difference with the other two groups was more evident. The present results are in accordance with Di Giulio et al. [16,39] and Drago et al. [16,39], who found that the topographical conformation of titanium surface was able to promote a significant influence on bacterial proliferation also after 72 h. The level of biofilm biomass (comprising bacterial cells/extracellular polymeric substances) was not significant at intergroup analysis, both at 24 and 48 h. The DAE group was characterized by a lower level of biofilm biomass when compared to the other two groups at 24 h, but the results were not statistically significant. 

Moreover, between 24 and 48 h, the biofilm biomass of the DAE group increased significantly, overlapping that of machined and single-etched surfaces but without significant differences.

The percentage of living cells in the DAE group was lower at 24 h when compared to other groups, but at 48 h, the results were similar to the single-etched group. 

These results may seem partially contrasting with our previous work, in which we compared the adhesion of *S. oralis* on three different surfaces: machined titanium, DAE, and peek [40]. In that case, the DAE surfaces appeared to interact with bacteria exactly like the machined surface, both at 24 and 48 h. 

It is fundamental to specify that, in that case, the DAE surfaces were subjected to different etching treatments [40].

Our hypothesis is that roughness is probably not the only factor that contributed to the antimicrobial activity of DAE surfaces. Pearson’s correlation has shown that biomass and CFU at 24 h were not significantly correlated with wettability (Table 1). On the contrary, the CFUs at 48 h were significantly correlated with these parameters. These data explain that in the first hours, a greater role could be played by the surface chemistry of the samples, and then, the topography of the surface increases its influence on the interaction with bacteria. The EDS analysis at 15 kV showed that the chemical composition of the samples was very similar; however, at this voltage, we have information about the composition of deeper layers. The same analysis at 5 kV permitted to obtain more data about the superficial layer. The higher oxygen percentage measured in the DAE samples could explain the higher bacterial inhibition.

However, other future studies on surface composition and electrostatic interactions should try to explain why two surfaces with a very similar Ra were characterized by such a significant bacterial response. The final decontamination process of the discs could play a fundamental role in leaving functional groups on the surfaces that could interfere with bacteria and cell interactions.

Xie et al. [41] have hypothesized that the treatment of sandblast-free double-etched titanium could leave reactive oxygen species (ROS) on the titanium surfaces, which could exert an antibacterial effect; so, the different performances of the three surfaces analyzed could depend on the different ability of the superficial treatment to leave free oxygen atoms on the titanium surface. 

## 4. Materials and Methods

### 4.1. Titanium Surfaces Preparation

This in vitro study was conducted using titanium discs, grade IV (Resista, Omegna (VB), Italy), 5 mm in diameter, and 2 mm in thickness. The process of disc production involved: washing with surfactants in a vacuum washing machine, combined with ultrasonic cavitation and a pickling process in hydrofluoric acid with neutralizer for final buffering. Three different surfaces were analyzed:

- Group 1: IV-grade titanium discs with a machined surface.
- Group 2 (Single-Etched Group): IV-grade titanium discs with surface treated by acid-etching. The discs were subjected to a single-etching process with subsequent treatment with nitric acid, hydrochloric acid, hydrofluoric acid, and final neutralizing buffer.
- Group 3 (DAE Group): IV-grade titanium discs with surface treated by double acid-etching. The discs were subjected to a double-etching process (DAE) with a mixture of nitric acid, hydrochloric acid, hydrofluoric acid, and final neutralizing buffer.

Then, all samples were washed in a basic solution with ultrasonic cavitation and decontaminated in a cold argon plasma reactor.

A total of 179 titanium discs were used in this in vitro study. This study is in compliance with the appropriate EQUATOR guidelines, the Standards for Reporting Qualitative Research SRQR [42].

**Figure 8:**
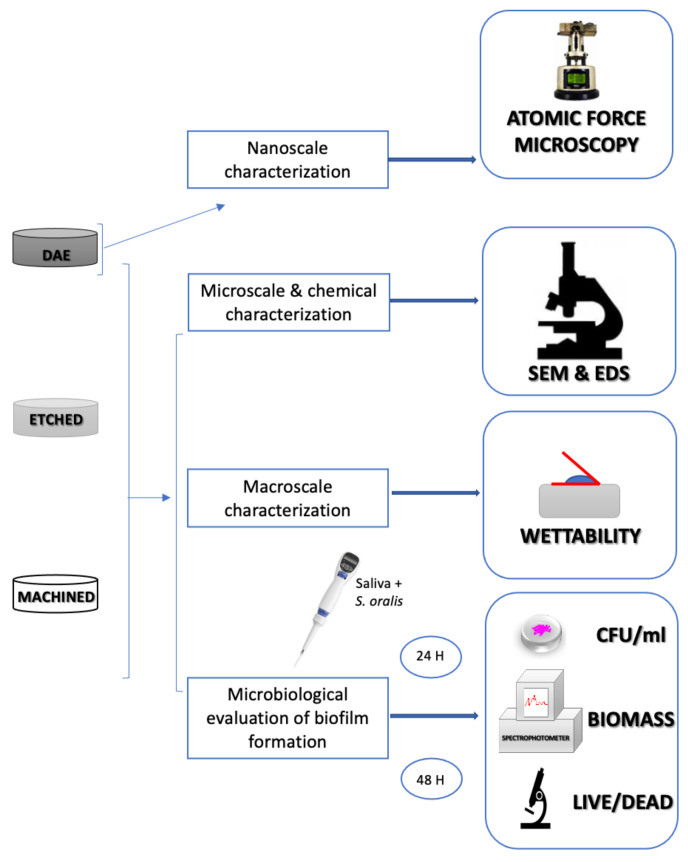
Schematic diagram illustrating the workflow of this study.

The flowchart of the experimental steps is shown in Figure 8.

All titanium discs were placed in 75% ethanol for 60 min, dried in a sterilized clean bench, and then irradiated on both sides with ultraviolet light for 30 min. A one-hour waiting period was observed after sterilization to allow the surfaces to equilibrate, as UV exposure has been shown to increase surface free radicals within the protective oxide layer on titanium [43]. Then, the sterile specimens were placed in 96-well polystyrene microtiter plates and, prior to each experiment, inoculated for 2 h in saliva at 37 °C in a shaking incubator with slight agitation to allow protein pellicle formation.

### 4.2. Scanning Electron Microscopy (SEM)

The samples were mounted on appropriate stubs with conductive glue and gold-coated (Emitech K550, Emitech Ltd., Ashford, Kent, UK) before observation, at different magnifications, by a Philips XL20 (Philips Inc., Eindhoven, The Netherlands) scanning electron microscope at 30 kV, equipped with an Edx microanalysis (EDS). Five discs for each group were observed. The percentage of porosity was measured by SEM observation at 480× of ETCHED and DAE samples, using ImageJ 1.52q for Mac OS X (USA), as previously described [44]. Ten samples for each group were observed, and the mean values (+/- standard deviation) were considered for the statistical analysis.

### 4.3. Energy-Dispersive X-ray Spectroscopy (EDS)

A Phenom ProX scanning electron microscope (Phenom-World BV, Eindhoven, the Netherlands) was used with the Element Identification (EID) package (Phenom ProSuite Software, Phenom-World B.V., The Netherlands) to characterize the superficial chemical composition of the samples. The following Field Of View (FOV) parameters were adopted: 179 µm; Mode: Map; detector: full Backscatter electron detector (BSD). The analysis was performed both at 15 and 5 kV of voltage. 

Three discs were observed for each group.

### 4.4. Atomic Force Microscopy (AFM)

Atomic force microscopy (AFM) was used to characterize the average nano-roughness (Ra) of DAE discs (group 3). 

The ScanAsyst technique was used for the atomic force microscopy (Bruker) observations with a scan size of 10 µm × 10 µm and a RTESPA-300 probe. The software Nanoscope was used to analyze images and for 3D reconstruction. The roughness average (Ra), which is the arithmetic mean of the absolute values of the height of the surface profile, was considered for the statistical analysis. The AFM observations were performed only in group 3, in order to verify the superficial topography of these discs that were not previously described in the literature. Ten samples were observed, and the mean values (+/− standard deviation) were considered for the statistical analysis.

### 4.5. Measurement of Wetting Properties

The sessile drop method was used to measure the wetting properties of the groups, as previously described [15]. A Nikon D90 DSLR camera (Nikon Corporation, Tokyo, Japan) with an 18−105-mm lens was used to photograph the samples (Figure 6). The water contact angle and the wetted area were measured using ImageJ 1.52q for Mac OS X (USA).

### 4.6. Microbial Strain

*Streptococcus oralis* CH 05, clinical strain, was used for this experimental study. The strain, stored at −80 °C, was recovered in Brain Heart Infusion broth (BHI, Oxoid, Milan, Italy) overnight at 37 °C under anaerobic condition. Then, the broth culture was diluted 1:10 in the same medium, refreshed for 2 h at 37 °C in a shaking thermostatic water bath (160 rpm), and standardized using a spectrophotometer (Eppendorf, Milan, Italy) to optical density OD_600_ = 0.12, corresponding to a concentration of 9 × 10^6^ CFU/mL. This bacterial suspension was used for the experiment. 

#### 4.6.1. Saliva Collection

Human saliva was taken from healthy volunteers with age > 18 years, as previously described [45,46,47]. The collection and use of saliva were approved by the Ethics Committee of University “G. d’Annunzio”, Chieti-Pescara, Italy (approval code SALI, N. 19 of the 10 September 2020). 

The inclusion criteria were: the absence of oral disease (caries or periodontitis); dental care not in progress; no antibiotics for three months prior to the beginning of the study. Samples were mixed, centrifuged at 16,000× *g* for 1 h at 4  °C, and filtered through a low-protein binding filter (pore sizes of 0.8, 0.45, and 0.2 μm) to remove microorganisms. Saliva was considered to be sterile if no growth could be detected in both aerobic and anaerobic atmospheres for 24–48 h at 37 °C [48]. Sterile saliva was stored at −20 °C and processed within two days.

#### 4.6.2. Biofilm Development

The discs coated with saliva were inoculated with 200 µL of *S. oralis* CH 05 bacterial suspension and incubated at 37 °C for 24 and 48 h under anaerobic condition. Negative controls, consisting of non-inoculated titanium discs, were also prepared. Then, microbial suspensions were carefully removed, and the samples were washed three times with Phosphate-buffered saline (PBS) to remove non-adherent bacteria. Each type of disc after 24 and 48 h of incubation was analyzed for:

(i) The CFU count for the quantification of cultivable cells.
(ii) The biofilm mass evaluation by Hucker’s crystal violet staining method.
(iii) The cell viability by LIVE/DEAD staining.

The microbiologic experiments were conducted in triplicate on each type of disc.

#### 4.6.3. Determination of Colony-Forming Units (CFUs)

After cultivating for 24 and 48 h, the adhered viable cells were washed with PBS to remove unattached cells. The discs were placed in a sterile test tube containing 1 mL PBS. Then, each test tube was placed into a 4- kHz ultrasonic cleaning water bath (Euronda, Italy) for 4 min followed by vortex mixing for 2 min to remove the bacteria attached on the surface of each disc.

Microscopic observations through viable staining prior to plating confirmed that the microbial suspension consisted of a mixture of single, viable microbial cells. Then, selected 10-fold dilutions were plated on Tryptic Soy Agar (TSA) plates and incubated overnight at 37 °C, followed by counting of CFU/mL. According to the above results, the number of adhered viable bacteria on the surface of the specimens was calculated to evaluate *S. oralis’* ability to colonize the different titanium surfaces. For this detection, 18 discs (3 for each sample surface and 3 control negative) were analyzed in triplicate for a total of 54 discs.

#### 4.6.4. Biofilm Biomass Assay

*S. oralis* CH 05 biofilm-forming ability was quantified by crystal violet staining. After 24 and 48 h of incubation, the discs were washed three times with PBS, fixed by air drying, stained with crystal violet 0.1% (Sigma-Aldrich, Milan, Italy) for 1 min, washed with PBS, and eluted with ethanol for reading. After 10 min, the samples were removed and the biofilm formation was quantified by measuring absorbance at 570 nm with a microplate reader (SAFAS, Munich, Germany).

The absorbance of the eluted stain is proportional to the concentration of biofilm biomass formed on the sample surface.

For this detection, 18 discs (3 for each sample surface and 3 control negative) were analyzed in triplicate for a total of 54 discs.

#### 4.6.5. Cell Viability Assay

For the evaluation of cell viability, the biofilm on disc surfaces was examined with a BacLight LIVE/DEAD Viability Kit (Molecular Probes, Invitrogen-Thermo Fisher Scientific, Milan, Italy). SYTO 9 stains viable cells with a green, fluorescent signal, and propidium iodide stains cells with impaired membrane activity in red. Then, attached bacteria were washed with PBS and stained for 15 min at room temperature in the dark, as indicated by the manufacturer. The images observed with fluorescent Leica 4000 DM microscopy (Leica Microsystems, Milan, Italy) were recorded at an emission wavelength of 500 nm for SYTO 9 (green fluorescence) and of 635 nm for propidium iodide (red fluorescence). The enumeration was performed by three blinded microbiologists by using an image analysis software LEICA QWin, (Version 3, Leica Microsystems, Milan, Italy) through the examination of at least 10 random fields of view each. 

For this detection, 9 discs (3 for each sample surface) were analyzed in triplicate for a total of 27 discs.

### 4.7. Statistical Analysis

Statistical analysis was performed using SPSS for Windows version 21 (IBM SPSS Inc., Chicago, IL, USA), analysis of variance (ANOVA) and the least significant difference (LSD) test were used to compare the parameters analyzed in the study for intra- and inter-group analysis. Data were analyzed using linear regression and descriptive statistics. T-test analysis was used to compare the percentage of porosity in the ETCHED and DAE samples. Pearson’s correlation was used to assess the relationship between the material features of the samples and the microbiological results. *p*-values less than 0.05 were considered significant.

## 5. Conclusions

This study demonstrated that double acid treatment on titanium discs characterized a superficial nano-roughness, increased hydrophilicity, and the oxygen composition of the superficial layer, and promoted a remarkable decrease in *S. oralis* biofilm formation in respect to machined and single-etched titanium. Within the limits of this study, DAE can be considered a promising treatment to counteract a pioneer microorganism colonization, such as *S. oralis.*

The best performance of DAE in terms of inhibition of *S. oralis* adhesion can influence biofilm/plaque formation and then decrease the risk of peri-implant diseases. 

Future research will need to take steps to more accurately recapitulate the interaction between DAE surface and biofilm, in vitro, and in vivo.

The current study opens up the possibility of a new type of surface which presents promising features for clinical applications.

## Figures and Tables

**Table 1:** The chemical composition (atomic percentage and weight concentration, ± standard deviation) of the samples analyzed by EDS at 5kV. ANOVA analysis and the least significant difference (LSD) post hoc test found significant differences (*p* < 0.05) between machined and DAE for all parameters investigated.

		Titanium		Oxygen	
		at %	wt%	at %	wt%
MACHINED	Mean	89.380	96.203	10.347	3.723
	St. Deviation	0.553	0.211	0.523	0.203
	Maximum	89.850	96.380	10.930	3.950
	Minimum	88.770	95.970	9.920	3.560
ETCHED	Mean	87.967	95.640	11.893	4.323
	St. Deviation	0.445	0.177	0.419	0.166
	Maximum	88.370	95.800	12.340	4.500
	Minimum	87.490	95.450	11.510	4.170
DAE	Mean	86.123	94.900	13.693	5.050
	St. Deviation	1.780	0.723	1.761	0.713
	Maximum	88.000	95.660	15.330	5.720
	Minimum	84.460	94.220	11.830	4.300

**Table 2:** The values of the Pearson’s correlation of the parameters analyzed in the study. * The correlation is significant at *p* < 0.05 (two-tails).

			% POROSITY	WCA	WETTED AREA
24 h	CFU	Pearson’s Correlation	−0.424	−0.277	0.162
		Sig. (Two-tails)	0.063	0.471	0.677
	BIOMASS	Pearson’s Correlation	−0.171	0.648	−0.629
		Sig. (Two-tails)	0.497	0.059	0.069
48 h	CFU	Pearson’s Correlation	−0.415	0.778 *	−0.737 *
		Sig. (Two-tails)	0.077	0.013 *	0.023 *
	BIOMASS	Pearson’s Correlation	0.133	−0.289	0.342
		Sig. (Two-tails)	0.666	0.45	0.368
